# A New Fault Feature Extraction Method for Rotating Machinery Based on Multiple Sensors

**DOI:** 10.3390/s20061713

**Published:** 2020-03-19

**Authors:** Feng Miao, Rongzhen Zhao, Xianli Wang, Leilei Jia

**Affiliations:** 1 School of Physical and Electrical Information, Luoyang Normal University, Luoyang 471022, China; mrx827@163.com (X.W.); jll1503@163.com (L.J.); 2 Key Laboratory of Digital Manufacturing Technology and Application, The Ministry of Education, Lanzhou University of Technology, Lanzhou 730050, China; zhaorongzhen@lut.cn

**Keywords:** rotating machine, median filter, rotor, vibration signal

## Abstract

During the operation of rotating machinery, the vibration signals measured by sensors are the aliasing signals of various vibration sources, and they contain strong noises. Conventional signal processing methods have difficulty separating the aliasing signals, which causes great difficulties in the condition monitoring and fault diagnosis of the equipment. The principle and method of blind source separation are introduced, and it is pointed out that the blind source separation algorithm is invalid in strong pulse noise environments. In these environments, the vibration signals are first de-noised with the median filter (MF) method and the de-noised signals are separated with an improved joint approximate diagonalization of eigenmatrices (JADE) algorithm. The simulation results found here verify the effectiveness of the proposed method. Finally, the vibration signal of the hybrid rotor is effectively separated by the proposed method. A new separation approach is thus provided for vibration signals in strong pulse noise environments.

## 1. Introduction

In the process of rotating machinery operation, the vibration signals measured by vibration sensors are often composed of the vibrations of multiple components [1,2]. Elucidating how to analyze, process, and identify these signals are very important for judging the working state of rotating machinery and fault diagnosis [3]. It is very difficult to analyze and process these sensor signals directly, which is bound to cause a lot of difficulties in mechanical condition monitoring and fault diagnosis [4]. The traditional modern signal processing method is obviously insufficient for vibration signals with multiple overlaps for rotating machinery [5,6,7,8]. 

In recent years, the development of digital signal processing technology has changed rapidly, and a large number of methods are used at present for the extraction and noise reduction of rotation fault signals. The empirical mode decomposition (EMD) proposed by Norden E. Huang et al. [9] is a non-stationary signal analysis method that can find the hidden feature information in a signal, and it is widely used in the fault extraction and noise reduction of rotating machinery [10,11,12]. The minimum entropy deconvolution [13,14] designs the optimal filter to eliminate the random noise in the bearing impact signal under the condition of maximizing the kurtosis value. The adaptive filter [15,16] needs to introduce an additional noise signal and extract the bearing fault impact signal by designing an optimal filter. According to the characteristics of bearing fault vibration and impact, the matching trace [17,18] defines atoms to disassemble the vibration signal and extract the impact characteristic components. In mathematical morphology analysis [19,20], the pre-defined structure operator is used to carry out corrosion, expansion, opening, and closing operations on the signal, such as to suppress the noise and extract the characteristic signal. Cyclostationary signal analysis [21,22] relies on the cyclostationary characteristics of the bearing fault impulse signal to design a filter to eliminate random noise. A Wiener filter [23,24] is used to eliminate the stationary random noise in the fault impulse signal. A wavelet transform [25,26] first decomposes the vibration signal into different frequency bands, then defines the threshold to eliminate the noise components and reconstructs the characteristic signal. A Kalman filter [27,28] first establishes the vibration state model and signal observation model of rolling bearing, then iteratively eliminates the noise in the signal. Stochastic resonance [29,30] uses noise to enhance the fault characteristic component of the bearing vibration signal. All of the above mentioned methods are universal, can effectively eliminate background noise and interference components, and extract fault signals in specific environment, but they are not suitable for complex interference situations, and especially when the interference components in vibration signals are similar to fault signals, it is difficult to distinguish them by the above method, let alone eliminating interference components and extract fault signals. Blind source separation (BSS) technology can realize the separation of multiple aliased signals [1,31]. Meanwhile, blind source separation is not affected by the time and frequency overlap of source signals, and the separated output signal will not lose the weak feature information in the source signal.

So far, many effective and distinctive blind source separation algorithms have been constructed. Typical algorithms include fast fixed-point [32] algorithms, natural gradient [28] algorithms, second-order blind identification (SOBI) [33] algorithms, equivalation adaptive separation via independence (EASI) [34] algorithms, and joint approximate diagonalization of eigenmatrices (JADE) [35] algorithms. When separating the noiseless mixed signals, these algorithms all show good separation performance. However, when separating signals with strong noise, there will be a lot of errors, even when the signal-to-noise ratio is low, where there will be a completely wrong conclusion because these algorithms are derived without considering the noise model. During the operation of the machine, the vibration signal measured by the vibration sensor inevitably contains a signal noise. When the blind source separation algorithm is used to separate the mixed vibration signals directly, it may cause great errors or draw incorrect conclusions. Therefore, it is very important to reduce noise before the blind separation of mechanical vibration signals, such as improve the signal-to-noise ratio. Median filtering [36] is a kind of nonlinear filtering method which has a strong ability to suppress impulse noise and has the characteristics of preserving edge profile information. It has been widely used in signal processing, for instance, in suppressing impulse noise. In order to solve the problem of the fault feature extraction of rotating machinery under a strong impulse noise, a fault separation method combining median filtering (MF) and an improved JADE algorithm (MF-JADE) is proposed. First, the median filtering method is used to de-noise the aliasing signal under strong impulse interference, then the improved JADE algorithm is used to separate the signal after noise reduction.

The contents of the following sections are as follows: Section 2 introduces the multi-sensor test system model. Section 3 presents the median filter algorithm. Section 4 presents the JADE algorithm. Section 5 presents the blind source separation method of multi-fault vibration signals based on MF-JADE. Simulated and experimental verifications are conducted in Section 6 and Section 7. Finally, the conclusions and outlook are both given in Section 8.

## 2. Multi-Sensor Test System Model

**Figure 1:**
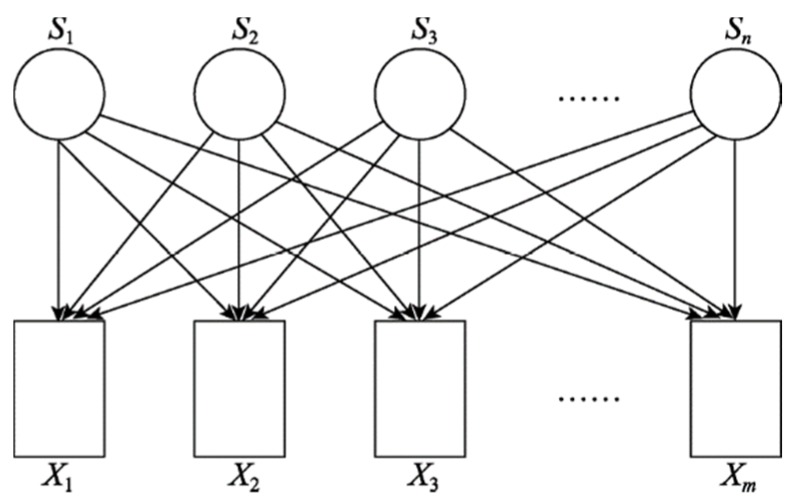
Diagram of a multi-source, multi-sensor testing procedure.

Generally, the signal measured by each sensor is a linear mixture of multiple original signals, as shown in Figure 1.

In Figure 1, the signals sent by n original signal sources ($s_{1},s_{2},s_{3},\ldots ,s_{n}$) are measured by m sensors and output observation signals ($x_{1},x_{2},x_{3},\ldots ,x_{n}$). In the actual test process, when multiple sensors are used for observation, the number of sensors are generally required to be not less than the number of signal sources, that is, m≥n. Assuming that the transmission is instantaneous and that the sensor receives a linear mixture of the original signal sources, the output of the i-th sensor is given as follows:

(1) $$x_{i}=\sum_{j=1}^{n}a_{ij}s_{j}(t)+v_{i}(t);i=1,2,3,\ldots ,m$$

where $a_{ij}$ is the mixing coefficient and $v_{i}(t)$ is the observation noise of the i-th sensor. The matrix form is given as follows:

(2) $$\left(\begin{array}{c} x_{1}\\ x_{2}\\ x_{3}\\ \vdots \\ x_{n} \end{array}\right)=\left(\begin{array}{ccc} a_{11} & \ldots & a_{1n}\\ \vdots & \vdots & \vdots \\ a_{m1} & \ldots & a_{mn} \end{array}\right)\left(\begin{array}{c} s_{1}\\ s_{2}\\ s_{3}\\ \vdots \\ s_{n} \end{array}\right)+\left(\begin{array}{c} v_{1}\\ v_{2}\\ v_{3}\\ \vdots \\ v_{n} \end{array}\right)$$

which is also written as follows:

(3) x(t)=As(t)+v(t)

In the formula, $A\in R^{m\times n}$ is an unknown rank full-rank mixed matrix, s(t) is an n-dimensional source vector, and v(t) is an additive noise vector, and its statistics are independent.

(4) y(t)=Wx(t)

The purpose of blind source separation is to find a separation matrix W, so that y(t)=Wx(t) is the optimal estimate of s(t).

A is a mixed matrix. To separate the source signal is to find the separation matrix U to make the following true:

(5) $$\overset{⌢}{S}(t)=UX(t)=UA\tilde{S}(t)$$

If

(6) UA=I

If I is a unit matrix, then $\overset{⌢}{S}(t)$ is an effective separation of $\tilde{S}(t)$.

Since blind source separation estimates the input signal according to only the observed signal, without any prior knowledge about the source signal, there are some uncertainties between the estimated input signal and the source signal, which are mainly reflected in the uncertainties in the estimation of the amplitude and order of the input signal. However, these two uncertainties do not affect the analysis of the signal, because most of the information of the signal is contained in the waveform rather than in the magnitude and order.

## 3. Median Filter

Median filtering belongs to a nonlinear sorting statistical filtering method. It sets a fixed length window to scan data by analyzing the distribution of sample data. In this process, the data in the window are sorted and the median value is taken as the output data after filtering at a certain point [37,38]. The median sequence obtained is the filtered signal. The sorting operation of this process can suppress the impulse noise in the signal well, but it retains the edge profile information of the original data and has a weak ability to suppress the stationary random noise superimposed linearly on the signal, therefore, the median filter is mainly used in signal processing which needs to suppress the impulse noise and retain the edge profile information.

The mathematical description of the one-dimensional signal median filter is given as follows. Suppose that a dataset consisting of k data is {x(1),x(2),…,x(k)}, let D be a filter window of length L=2N+1, where N is a positive integer. We define the dynamic sub-window as follows:

(7) $$W_{N}(m:n)=\{x(m,n-i);-N\leq i\leq N\}$$

We set 2N+1 data in the input window at the n-th time as {x(n−N),…,x(n),…,x(n+N)|n+N≤k}, where the output of the median filter is then defined as follows:

(8) $$s(n)=\left\{ \begin{array}{l} med[x(n-N),\ldots ,x(n+N)];m≺n\leq N-m\\ y(n);others \end{array}\right.$$

where med[•] denotes that the operation of the data in the window is arranged in an ascending order and then the median value is taken.

Scanning the samples with windows, the output sequence median s(n)(1≤n≤k−N+1) is the filtered signal. It can be seen that the superposition principle is no longer tenable at this time, so the median filter is a non-linear filtering method, and its ability to suppress smooth noise is weak.

The above process shows that the median filter is a neighborhood operation, which selects the median of the window sequence as the output calculation. In the output median sequence of dynamic window D, there is a white noise component of a Gaussian distribution that is superimposed linearly in the sequence, and it has a defect, that is, difficult to process the edge signal. Therefore, this filtering method can protect the details of the linear superimposed stationary random noise and the ability to suppress the Gaussian white noise is weak.

## 4. Independent Component Analysis Based on JADE

If the vibration source signals are statistically independent of each other, the process of blind source separation is typically carried out to find the independent elements in the observed mixed signals. The key step here is to find the mixed matrix A or the separated matrix U.

### 4.1. Hybrid Matrix A Estimation Based on the Fourth-Order Cumulant

The JADE algorithm is an algorithm based on a fourth-order cumulant. For n random variables $x_{1},\ldots x_{n}$, fourth-order cumulants are defined as follows:

(9) $$cum(x_{i},x_{j},x_{k},x_{l})=E[x_{i}x_{j}x_{k}x_{l}]-E[x_{i}x_{j}]E[x_{k}x_{l}]-E[x_{i}x_{k}]E[x_{j}x_{l}]-E[x_{i}x_{l}]E[x_{j}x_{k}]$$

where i,j,k,l=1,2,…,n. The elements of row i and column j corresponding to the fourth-order cumulant matrix can be expressed as follows:

(10) $${\left[C_{x}(M)\right]}_{i,j}=\sum_{k=1}^{n}\sum_{l=1}^{n}cum(x_{i},x_{j},x_{k},x_{l})m_{kl}$$

where $m_{kl}$ is the k,l element of any weight matrix M of order n×n. If the mean value of each variable in X is zero, according to Equations (9) and (10), the cumulant matrix of X can be expressed as follows:

(11) $$C_{X}(M)=E[(X^{T}MX)XX^{T}]-R_{X}tr(MR_{X})-R_{X}MR_{X}-R_{X}M^{T}R_{X}$$

where tr(·) is the trace of the matrix and $R_{X}$ is the covariance matrix of matrix X. It has been proven in [39] that the fourth-order cumulant matrix can be expressed as follows:

(12) $$C_{X}(M)=A\Delta (M)A^{T}$$

(13) $$\Delta (M)=Diag(\lambda_{1}a_{1}^{T}Ma_{1}\ldots \lambda_{n}a_{n}^{T}Ma_{n})$$

where $\lambda_{i}$ is the eigenvalue of $C_{X}(M)$, $a_{i}$ is the column vector of A, and $i=1,2,\ldots ,n;A=[a_{1},a_{2},\ldots ,a_{n}]$.

Take two n×n-order matrices $M_{1}$ and $M_{2}$, then, according to Equation (10), we can obtain the following:

(14) $$C_{X}(M_{1})=A\Delta (M_{1})A^{T}$$

(15) $$C_{X}(M_{2})=A\Delta (M_{2})A^{T}$$

$\Delta (M_{1})$ and $\Delta (M_{2})$ are diagonal matrices.

(16) $$G=C_{X}(M_{1})C_{X}^{-1}(M_{2})=(A\Delta (M_{1})A^{T}){\left(A\Delta (M_{2})A^{T}\right)}^{-1}=A\Delta A^{-1}$$

Here, $\Delta =\Delta (M_{1})\Delta {\left(M_{2}\right)}^{-1}$ is a diagonal matrix.

From Equation (10), it can be concluded that

(17) GA=AΔ

Therefore, the diagonal element of Δ can be regarded as the eigenvalue of G, and A is the eigenvector of G. Theoretically, finding the eigenvector of G is equivalent to finding the mixed matrix A.

### 4.2. Signal Whitening

The above discussion is based on the condition that matrix A is invertible and the source signals are statistically independent of each other. Therefore, in the actual situation, it is necessary to first add the central and whitening process to the observation signal to ensure that the conditions are established. Centralization is to replace $x_{i}(t),i=1,2,\ldots ,n$ with $x_{i}(t)-E[x_{i}]$, such that the observation sequence $x_{i}(t)$ becomes a zero mean sequence. Whitening is to remove the correlation between the components and ensure the statistical independence between the components. Without losing generality, if the matrix of observation variables after centralization is still set as $X(t)={\left[x_{1}(t),\ldots ,x_{n}(t)\right]}^{T},t=1,2,\ldots ,N$, where N is the number of observation points, then its covariance matrix can be expressed as follows:

(18) $$R_{X}=E[XX^{T}]=V\Sigma^{2}V$$

If V is the unitary matrix and Σ is the eigenvalue diagonal matrix of $R_{X}$, then the whitening transformation matrix Q can be expressed as follows:

(19) $$Q=\Sigma^{-1}V^{T}$$

The whitened signal can be expressed as follows:

(20) Z(t)=QX(t)

where the covariance matrix of which can be written as follows:

(21) $$R_{Z}=E[ZZ^{T}]=\Sigma^{-1}V^{T}V\Sigma^{2}V^{T}V\Sigma^{-1}=I$$

In order to realize whitening, we make H=QA, then

(22) $$Z(t)=H\tilde{S}(t)$$

In Section 4.1, the process of finding A based on X(t) has been transformed into the process of finding H based on the whitened matrix Z(t).

### 4.3. Joint Approximate Diagonalization

In practical calculation, because of the existence of numerical calculation error and interference noise, it is impossible to achieve complete diagonalization, only approximate diagonalization. It is impossible to find the optimal H value for any two matrices, i.e., $M_{1}$ and $M_{2}$, so the joint approximate diagonalization method is used instead. Based on the whitened signal Z(t), we take a p n×n-order arbitrary matrices $M_{1},\ldots M_{p}$. In order to meet the accuracy requirements, we generally take $p=n^{2}$. Next, we find $C_{z}(M_{i})(i=1,2,\ldots ,p)$ for each $M_{i}$, find a unitary matrix H, and make the following formula reach a minimum value:

(23) $$C(H)=\sum_{M_{i}}off[H^{T}C_{Z}(M_{i})H]$$

where off(·) is defined as the sum of squares of all nondiagonal elements of the matrix. The estimation of the independent elements of mixed signals can be expressed as follows:

(24) $$S(t)=H^{T}Z(t)$$

## 5. Blind Source Separation of Multi-Fault Vibration Signals Based on MF-JADE

Sensors are usually arranged on the X and Y axes of rotating machinery, the collected signals are transmitted, and the statistical independence of each signal is easily affected by the transmission time difference, noise, and so on. When blind source separation is performed, median filtering and whitening processing are required first.

### 5.1. Basic Steps

Step 1: Carry out median filtering and centralized processing for the fault signal data observed in each channel;

Step 2: Whiten the data with Equations (18)–(20) to get Z(t),t=1,2,…,N.

Step 3: Take p weight matrices for $M_{1},\ldots M_{p}$, and calculate the fourth-order cumulant matrix $C_{Z}(M_{i}),i=1,2,\ldots ,p$ of Z(t) according to Equation (11), where generally $p=n^{2}$.

Step 4: The matrix group $C_{Z}(M_{i})$ of Step 3 is jointly approximately diagonalized to minimize the optimization objective function (Equation (23)), and thus the unitary matrix H is obtained.

Step 5: Calculate the separation matrix $U=H^{T}Q$.

Step 6: Estimate the source signal according to Equation (5).

Step 7: Analyze the signal characteristics and conduct fault diagnosis.

### 5.2. Evaluation Index of Separation Effect

In order to quantitatively explain the effect of BSS, it is necessary to consider a variety of performance evaluation indices to reflect the error measurement between the separated signal and the original source signal from different aspects. In this paper, three independent meta-analysis evaluation indices, i.e., the correlation coefficient, $\rho_{i}$, secondary residual, VQM, and performance index, PI, are introduced. If $s_{i}$ is the i-th vibration source signal and $\overset{⌢}{s}$ is the separation signal corresponding to $s_{i}$, the correlation between $s_{i}$ and $\overset{⌢}{s}$ can be expressed as follows:

(25) $$\rho_{i}=\frac{\mathrm{cov}(s_{i},{\overset{⌢}{s}}_{i})}{\sqrt{\mathrm{cov}(s_{i},s_{i})\mathrm{cov}({\overset{⌢}{s}}_{i},{\overset{⌢}{s}}_{i})}}$$

where cov(·) represents variance. When the signal separated by the independent component analysis (ICA) algorithm is close to the corresponding source signal, the closer the value of $\left|\rho_{i}\right|$ to 1, the better the separation effect.

A calculation formula with an amplitude correction factor was adopted for the secondary residual, which can be expressed as follows:

(26) $$VQM=10\mathrm{lg}\frac{E[{\left|{\overset{⌢}{s}}_{i}(t)-rs_{i}(t)\right|}^{2}]}{E[{\left|rs_{i}(t)\right|}^{2}]}$$

The smaller the value of VQM, the better the separation effect. When the value is less than −23 dB, the separation effect is better.

By Equations (5) and (6), we let Φ=UA. Ideally it should be a unit array. Considering the uncertainty of the arrangement order of output vectors in the ICA method, Φ can be a matrix with only one element in each row and column. At this time, a source signal corresponds to a separate signal, which is an effective separation. PI is the index to measure the difference between the actual Φ matrix and the one-to-one correspondence requirements above. Its formula can be expressed as follows:

(27) $$PI=\frac{1}{n(n-1)}\sum_{i=1}^{n}\left\{(\sum_{k=1}^{n}\frac{\left|h_{ik}\right|}{\max_{j}\left|h_{ij}\right|}-1)+(\sum_{k=1}^{n}\frac{\left|h_{ki}\right|}{\max_{j}\left|h_{ji}\right|}-1)\right\}$$

where $h_{ij}$ is the (i,j) element of matrix Φ. The smaller the PI value, the better the separation effect.

In addition, the vibration signals generated by the friction and collision of fatigue-damaged parts must have certain periodic characteristics. Therefore, for rotating machinery, the frequency characteristics, such as the resonance frequency, are key factors to reflect the effectiveness of the separation signal, which also needs to be included in the evaluation index of the separation effect.

## 6. Simulations 

In order to verify the effectiveness of the algorithm, three periodic signals with different frequencies were used to simulate the vibration mixing caused by different rotating frequencies for machines. Generally, the vibration signal of a single rotating shaft can be simply regarded as the superposition of its rotating frequency and its double frequency. The expression of the source signal can be expressed as follows:

(28) $$s_{i}(t)=\sum_{k=1}^{2}A_{ki}\sin(2\pi kf_{i}t+\varphi_{ki})\;\left(i=1,2,3\right)$$

where $A_{ki}$ is the amplitude of the i-th source signal, $f_{i}$ is the frequency conversion of the i-th source signal, $kf_{i}$ is the k-times frequency conversion of the i-th source signal, *φ*_ki_ is the phase, and $A_{ki}$ and $\varphi_{ki}$ are randomly generated by the computer. Here, the $f_{i}$ values are 25, 50, and 75 Hz respectively. 

**Figure 2:**
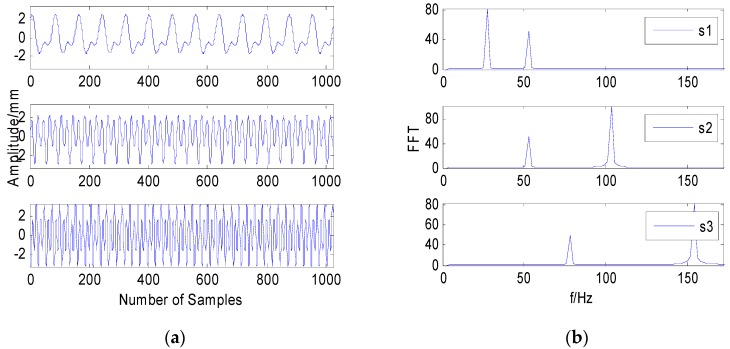

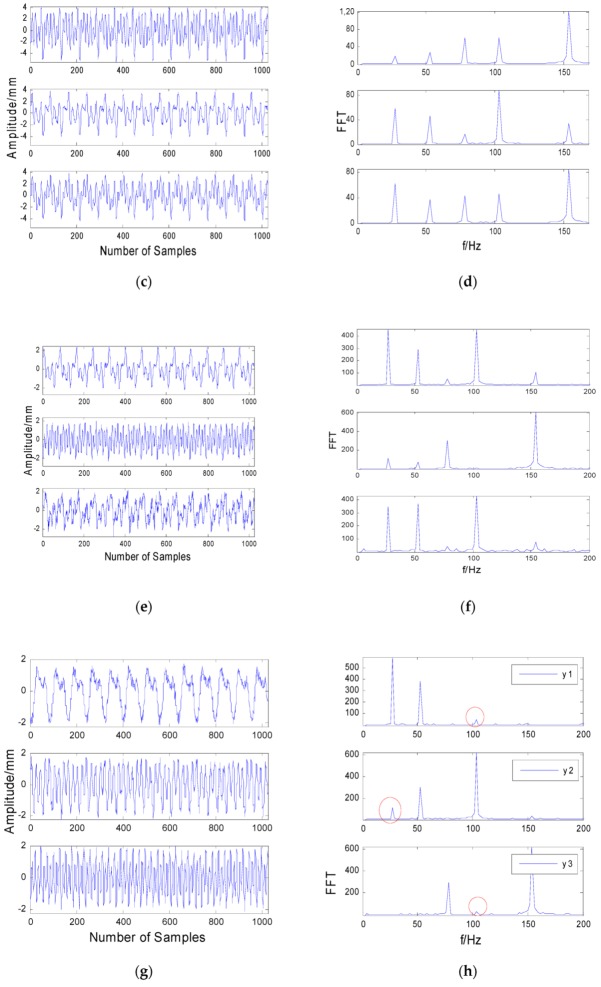

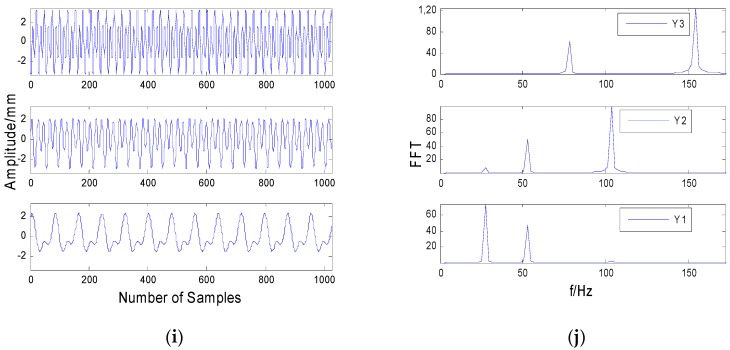
Simulation analysis of vibration signals with strong noises. (**a**)The source signals in the time domain. (**b**) The source signals in the frequency domain. (**c**) The signals mixed noises in the time domain. (**d**) The signals mixed noises in the frequency domain. (**e**) The separated signals with noise by joint approximate diagonalization of eigenmatrices (JADE). (**f**) The separated signals with noise by JADE. (**g**) The separated signals by MF-FastICA. (**h**) The separated signals by MF-FastICA. (**i**) The separated signals by the MF-JADE method. (**j**) The separated signals by the MF-JADE method.

Figure 2a,b shows the time domain waveform and spectrum of the source signals. After the source signal is mixed by a random matrix, Gaussian white noise and impulse noise with a 25 dB SNR are added, and the time-domain waveform and spectrum are shown in Figure 2c,d. The time domain waveform and spectrum obtained by the FastICA algorithm are shown in Figure 2e,f. Figure 2g,h show the time-frequency waveform based on MF-FastICA. Figure 2i,j show the time-frequency waveform based on MF-JADE.

It can be seen from Figure 2f that in the case of pulse signal interference, the source signal has not been separated, and there is a large error. This can show that the algorithm based on the noiseless model will produce a lot of errors in the separation of noisy data, even leading to incorrect results. 

Comparing Figure 2h,b, it can be seen that the corresponding relationship between the separated signal and the source signal is $y_{1}\to s_{1},y_{2}\to s_{2},y_{3}\to s_{3}$. It can be seen from Figure 2h that the separation method based on MF-FastICA has more frequency components marked by red circles. However, it is much better than the direct separation based on the FastICA method.

Comparing Figure 2j,b, it can be seen that the corresponding relationship between the separated signal and the source signal is $y_{1}\to s_{1},y_{2}\to s_{2},y_{3}\to s_{3}$. The separation and source signals only exist in the uncertainty of amplitude and sequence, which does not affect the identification of fault characteristics. In Figure 2j, the peaks of 75 Hz and 150 Hz are very obvious in the first figure, and other frequencies are basically suppressed, indicating that this signal has been well separated. The peaks of 50 Hz and 100 Hz are also very obvious in the second figure, although there are peaks of other frequencies. In the third figure, the peaks of 25 Hz and 50 Hz are also very obvious. The evaluation index values are shown in Table 1. As can be seen from Table 1, the method proposed in this paper is better than the traditional methods of FastICA and JADE.

The simulation results show that before the blind separation of vibration signals under the interference of impulse noise, the median filter method can effectively remove impulse noise, improve the signal-to-noise ratio, and effectively achieve the extraction of fault features.

## 7. Experiments 

**Figure 3:**
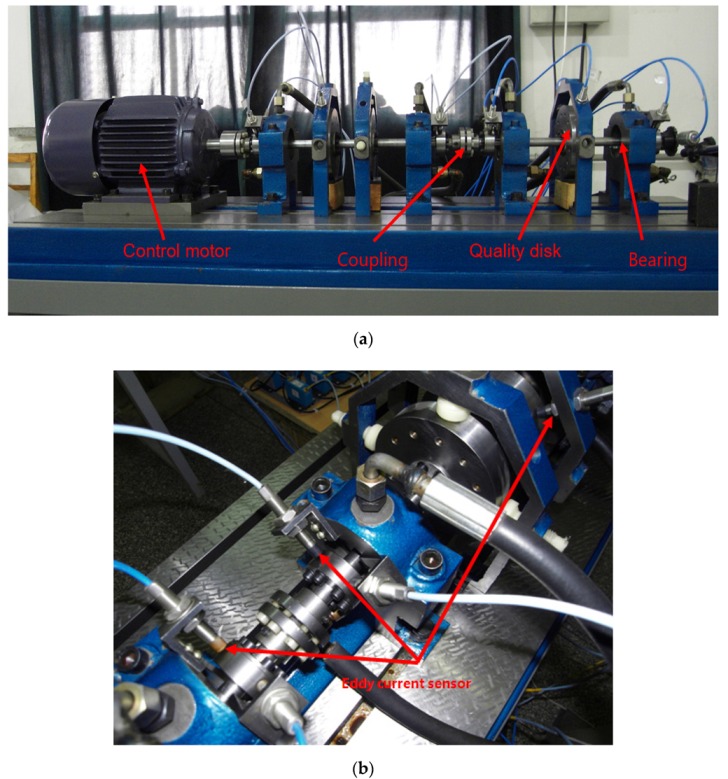

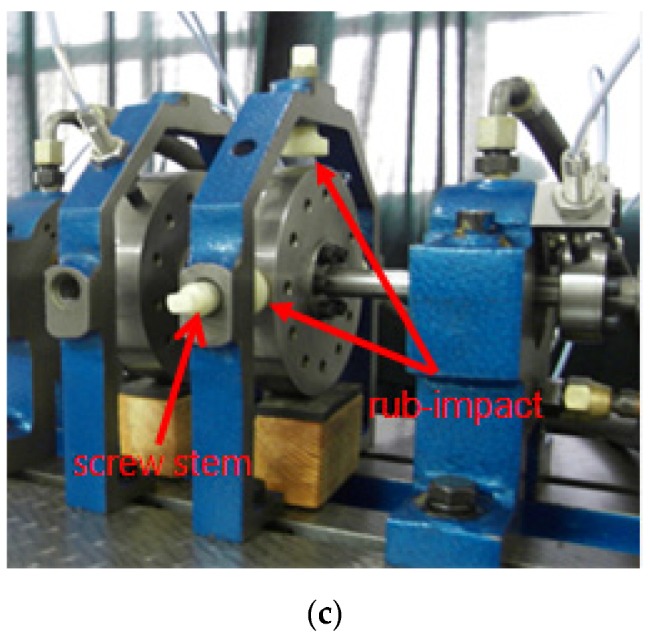
The two-span rotor-bearing rig. (**a**) The rotor testbed. (**b**) Installation positions of sensors. (**c**) Rotor rub test.

In order to verify the separation performance of the proposed algorithm for the measured mixed vibration signal, an experimental platform was built to analyze the measured mixed rotor vibration signal. Since there may be multiple potential source signals in the process of rotor rotation, such as the vibration signal of ball bearings, axial vibration signals, and noise signals from shafts, and since the sensor is measuring at the same time, the signal measured by the sensor is the mixed vibration signal. In order to satisfy the assumption that the number of sensors is greater than or equal to the number of source signals in blind source separation, five sensors were used in the experiment. The installation positions of the sensors are shown in Figure 3b. The rotating speed of the rotor was about 3200r/min and the sampling frequency was 5 KHz. Figure 3a shows the rotor test bench. The testbed was used to simulate the rub impact fault, and the simulated fault debugging part is shown in Figure 3c. 

**Figure 4:**
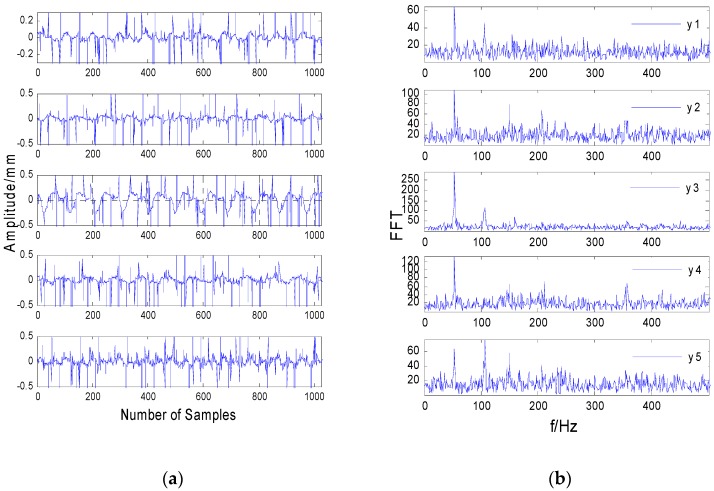
Time-frequency waveforms of rotor vibration signals. (**a**) The time-domain signals. (**b**) The frequency-domain signals.

**Figure 5:**
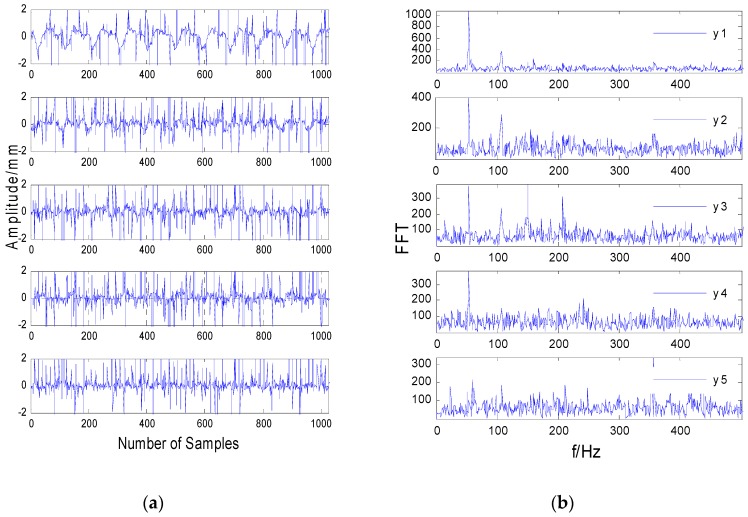
The signals separated by FastICA. (**a**) The time-domain signals. (**b**) The frequency-domain signals.

Figure 4a shows the time-domain vibration signals collected by the sensor in the case of a rub impact fault. In the case of a rub impact fault, the classic FastICA algorithm was directly used to separate the sampling signals. The time-domain waveform of the separated signal is shown in Figure 5a. Comparing Figure 4a and Figure 5a, it can be seen there is no obvious difference between the mixed signal measured by the actual rotor test bench and the separated signal in the time domain.

**Figure 6:**
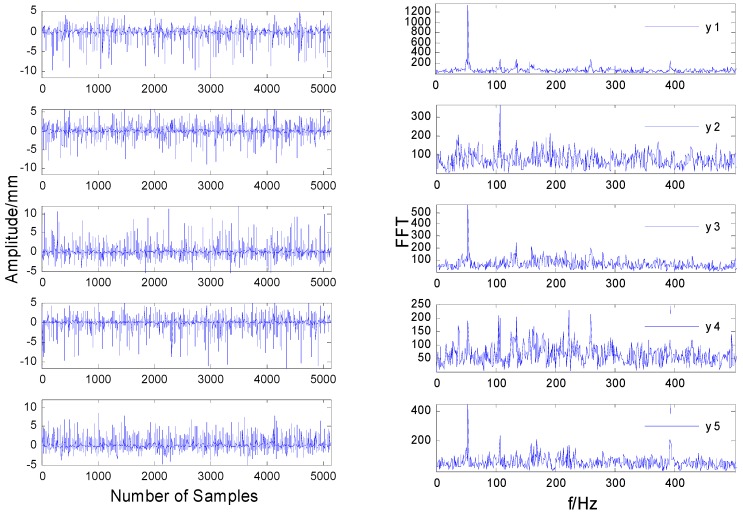
The separated signals by the JADE method. (**a**) The time-domain signals. (**b**) The frequency-domain signals.

**Figure 7:**
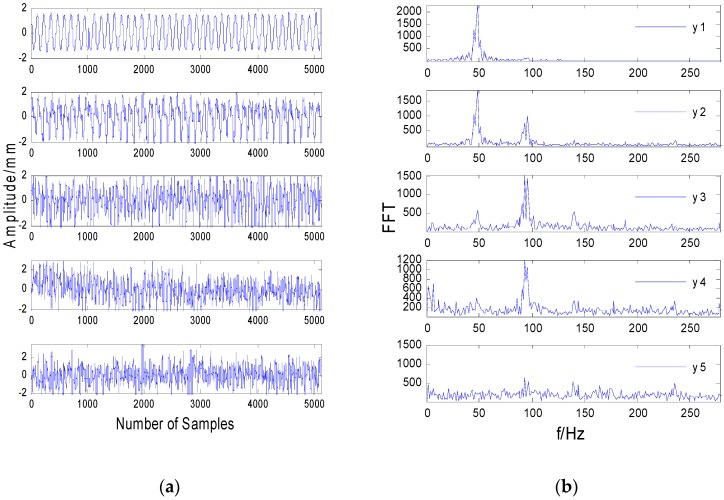
The separated signals by the MF-JADE method. (**a**) The time-domain signals. (**b**) The frequency-domain signal.

Figure 6a shows the time-domain vibration signals separated by the JADE algorithm. Figure 7a shows the time-domain vibration signals separated by median noise reduction and the JADE algorithm. Comparing Figure 4a and Figure 7a, it can be seen the impulse noise is well suppressed after median filtering.

In order to compare the complex vibration of the rotor before and after separation more intuitively, it is necessary to analyze the spectrum of each data signal before and after the separation and observe the different characteristics of the signal before and after the separation from the frequency domain. 

It can be seen from Figure 4b that most of the frequencies are submerged in the noise, and the frequencies are mixed, except that the frequencies in the third figure are not submerged by the noise. As shown in Figure 5b, the spectrum after direct separation still contains a lot of noise, and each frequency is not completely separated, which shows that the separation effect of the FastICA algorithm is significantly worse when the data contain strong impulse noise. Comparing Figure 5b and Figure 7b, it can be seen the spectral line of 100 Hz in Figure 5b is submerged by noise and cannot be identified, while the characteristic spectrum line of 100 Hz in Figure 7b is highlighted. This shows that the performance of the MF-JADE algorithm is better than that of direct separation when it is used to separate aliased signals in impulsive noise environments, where it can effectively suppress noise signals and highlight the periodic signals.

In the first figure in Figure 7b, it can be seen the frequency of 50 Hz is highlighted while the other frequencies are suppressed. Since the power frequency used in daily life is 50 Hz, it can be determined that the signal is a power frequency signal. The second figure in Figure 7b has two frequency values, one is 50 Hz, which can be calculated as the rotor’s rotating frequency, and the other is 100 Hz, which is two-fold frequency. Since the amplitude of the first-fold frequency is greater than that of the second-fold frequency, it can be seen that the signal is the unbalanced rotor fault signal. From the third and fourth figures in Figure 7b, it can be seen that the amplitude of the first octave is less than that of the second octave and that there are other octave spectral lines, so it can be seen that the signal is the rotor rub impact fault signal. From the fifth figure in Figure 7b, it can be seen that the frequency of this figure is distributed over the entire frequency band. From Figure 7a, it can be seen that the signal is random in the time domain. Combining these two points, it can be determined that this signal is a noise signal.

Comparing Figure 5 and Figure 7, the improved method proposed in this paper can effectively separate the rub impact fault and mass disk imbalance fault caused by the rub impact and the noise signal. However, using the classical FastICA and JADE separation method, we can only separate the 50 Hz power frequency signal of the rotor system, as shown in Figure 5 and Figure 6.

## 8. Conclusions

In order to solve the problem of fault feature extraction for rotating machinery in a strong impulse noise environment, a fault separation method combining the median filter and an improved JADE algorithm (MF-JADE) has been proposed here. Through simulation and an experimental study of the vibration signal separation of a hybrid rotor, the following conclusions may be made:

1. Blind separation of the observation signal with strong impulse noise was carried out directly, and the error of the separation result was large, where even an incorrect result could be obtained.
2. The median filtering method can effectively remove the impulse noise signal without losing the useful components of the original signal, improve the signal-to-noise ratio, and provide precondition for the accurate realization of blind separation.
3. For the measured signal, although the independence assumption of blind source separation is not strictly true, the MF-JADE algorithm is still effective in the actual vibration signal separation.
4. The combination of a median filtering method and a blind source separation algorithm provides a new method for the separation of aliased signals in strong impulse noise environments.

## Figures and Tables

**Table 1:** Evaluation indices comparison for blind source separation.

Algorithm	$s_{i}$	$\rho_{i}$	VQM/dB	PI
Fast-ICA	$s_{1}$	0.455	1.612	
	$s_{2}$	0.523	−2.765	3.415
	$s_{3}$	0.632	−3.316	
JADE	$s_{1}$	0.579	−3.422	
	$s_{2}$	0.814	−8.563	3.413
	$s_{3}$	0.611	−2.013	
MF-FastICA	$s_{1}$	0.915	−14.213	
	$s_{2}$	0.921	−13.612	0.965
	$s_{3}$	0.936	−15.518	
MF-JADE	$s_{1}$	0.998	−23.612	
	$s_{2}$	0.986	−20.965	0.311
	$s_{3}$	0.997	−22.953	

## References

[1] Fu W., Tan J., Zhang X., Chen T., Wang K. (2019). Blind Parameter Identification of MAR Model and Mutation Hybrid. GWO-SCA Optimized SVM for Fault Diagnosis of Rotating Machinery. Complexity.

[2] Glowacz A. (2019). Fault diagnosis of single-phase induction motor based on acoustic signals. Mech. Syst. Signal Process..

[3] Xia M., Li T., Xu L., Liu L., De W. (2018). Fault Diagnosis for Rotating Machinery Using Multiple Sensors and Convolutional Neural Networks. IEEE-Asme Trans. Mechatron..

[4] Jiang X., Wang J., Shi J., Shen C., Huang W., Zhu Z. (2019). A coarse-to-fine decomposing strategy of VMD for extraction of weak repetitive transients in fault diagnosis of rotating machines. Mech. Syst. Signal Process..

[5] Song L., Wang H., Chen P. (2018). Vibration-Based Intelligent Fault Diagnosis for Roller Bearings in Low-Speed Rotating Machinery. IEEE Trans. Instrum. Meas..

[6] Shao H., Jiang H., Zhao H., Wang F. (2017). A novel deep autoencoder feature learning method for rotating machinery fault diagnosis. Mech. Syst. Signal Process..

[7] Shao H., Jiang H., Zhao H., Wang F. (2017). An enhancement deep feature fusion method for rotating machinery fault diagnosis. Knowl. Based Syst..

[8] Rostaghi M., Ashory M.R., Azami H. (2019). Application of dispersion entropy to status characterization of rotary machines. J. Sound Vib..

[9] Lun D.P.K., Chan Y.-H. (2018). Robust Single-Shot Fringe Projection Profilometry Based on Morphological Component Analysis. IEEE Trans. Image Process..

[10] Conci A., Rodrigues E.O., Liatsis P. (2018). Morphological classifiers. Pattern Recognit..

[11] Huang W., Liu J. (2020). Robust Seismic Image Interpolation with Mathematical Morphological Constraint. IEEE Trans. Image Process..

[12] Legaz-Aparicio A.-G., Verdu-Monedero R., Angulo J. (2018). Adaptive morphological filters based on a multiple orientation vector field dependent on image local features. J. Comput. Appl. Math..

[13] Lin H., Hu Y., Chen S., Yao J., Zhang L. (2019). Fine-grained classification of cervical cells using morphological and appearance based convolutional neural networks. IEEE Access.

[14] Luo B., Zhang L. (2014). Robust autodual morphological profiles for the classification of high-resolution satellite images. IEEE Trans. Geosci. Remote Sens..

[15] Lv Z., Zhang P., Benediktsson J., Shi W. (2014). Morphological profiles based on differently shaped structuring elements for classi fication of images with very high spatial resolution. IEEE J. Sel. Top. Appl. Earth Obs. Remote Sens..

[16] Ma W., Wan Y., Li J., Zhu S., Wang M. (2019). An automatic morphological attribute building extraction approach for satellite high spatial resolution imagery. Remote Sens..

[17] Naderi H., Fathianpour N., Tabaei M. (2019). MORPHSIM: A new multiple-point pattern-based unconditional simulation algorithm using morphological image processing tools. J. Pet. Sci. Eng..

[18] Nair V., Ram P.G.K., Sundararaman S. (2019). Shadow detection and removal from images using machine learning and morphological operations. J. Eng..

[19] Ritchey T. (2018). General morphological analysis as a basic scientific modelling method. Technol. Forecast. Soc. Chang..

[20] Salazar-Colores S., Cabal-Yepez E., Ramos-Arreguin J., Botella G., Ledesma-Carrillo L., Ledesma S. (2019). A Fast Image Dehazing Algorithm Using Morphological Reconstruction. IEEE Trans. Image Process..

[21] Tong W., Li H., Chen G. (2020). Blob detection based on soft morphological filter. IEICE Trans. Inf. Syst..

[22] Wang Z. (2020). A new clustering method based on morphological operations. Expert Syst. Appl..

[23] Xue B., Hong H., Zhou S., Chen G., Li Y., Wang Z., Zhu X. (2019). Morphological Filtering Enhanced Empirical Wavelet Transform for Mode Decomposition. IEEE Access.

[24] Yan X., Liu Y., Jia M. (2019). A feature selection framework-based multiscale morphological analysis algorithm for fault diagnosis of rolling element bearing. IEEE Access.

[25] Zhang B., Hu Q., Wei L., Li S. (2018). Improved morphological filtering algorithm of interferograms. Xi Tong Gong Cheng Yu Dian Zi Ji Shu/Syst. Eng. Electron..

[26] Zhang X., Tang C., Zhou R., Lei W. (2018). Adaptive Morphological Filtering Algorithm with Applications in Bearing Fault Diagnosis. Hsi-Chiao Tung Ta Hsueh/J. Xi’an Jiaotong Univ..

[27] Benko G., Juhasz Z. (2019). GPU implementation of the FastICA algorithm. Proceedings of the 42nd International Convention on Information and Communication Technology, Electronics and Microelectronics, MIPRO 2019.

[28] Chen L., Gan S., Zhang L., Wang G. (2017). Nonlinear blind source separation algorithm based on spline interpolation and artificial bee colony optimization. Tongxin Xuebao/J. Commun..

[29] Li C., De Oliveira J.V., Cerrada M., Cabrera D., Sanchez R., Zurita G. (2019). A Systematic Review of Fuzzy Formalisms for Bearing Fault Diagnosis. IEEE Trans. Fuzzy Syst..

[30] Zhang H., Ma J., Jing J., Li P. (2019). Fabric defect detection method based on improved fast weighted median filtering and K-means. J. Text. Res..

[31] Lu S., He Q., Wang J. (2019). A review of stochastic resonance in rotating machine fault detection. Mech. Syst. Signal Process..

[32] Aldhahab A., Mikhael W.B. (2018). Face Recognition Employing DMWT Followed by FastICA. Circuits Syst. Signal Process..

[33] Fantinato D., Duarte L., Deville Y., Attux R., Jutten C., Neves A. (2019). A second-order statistics method for blind source separation in post-nonlinear mixtures. Signal Process..

[34] Huang J., Sun J. (2018). Sampling Adaptive Learning Algorithm for Mobile Blind Source Separation. Wirel. Commun. Mob. Comput..

[35] Li Y., Nie W., Ye F., Wang Q. (2017). A complex mixing matrix estimation algorithm in under-determined blind source separation problems. Signal Image Video Process..

[36] Zhou Y., Li S., Zhang D., Chen Y. (2018). Seismic noise attenuation using an online subspace tracking algorithm. Geophys. J. Int..

[37] Yang J., Zhang Y., Yin W. (2009). An Efficient Tvl1 Algorithm for Deblurring Multichannel Images Corrupted by Impulsive Noise. Siam J. Sci. Comput..

[38] Babaee M., Tung D.D., Rigoll G. (2018). A deep convolutional neural network for video sequence background subtraction. Pattern Recognit..

[39] Qin Z., Yi Y.-Q., Lin Y.-P. (2008). Robust watermark based on JADE algorithm. Tien Tzu Hsueh Pao/Acta Electron. Sin..

